# Gut Microbiota-Testis Axis: FMT Mitigates High-Fat Diet-Diminished Male Fertility via Improving Systemic and Testicular Metabolome

**DOI:** 10.1128/spectrum.00028-22

**Published:** 2022-04-21

**Authors:** Yanan Hao, Yanni Feng, Xiaowei Yan, Liang Chen, Xiangping Ma, Xiangfang Tang, Ruqing Zhong, Zhongyi Sun, Manjree Agarwal, Hongfu Zhang, Yong Zhao

**Affiliations:** a State Key Laboratory of Animal Nutrition, Institute of Animal Sciences, Chinese Academy of Agricultural Sciences, Beijing, People’s Republic of China; b College of Science, Health, Engineering and Education, Murdoch University, Perth, Australia; c College of Veterinary Medicine, Qingdao Agricultural University, Qingdao, People’s Republic of China; d Urology Department, Shenzhen university general hospital, Shenzhen, People’s Republic of China; The Pennsylvania State University

**Keywords:** obesity, HFD, AOS, A10-FMT, male infertility, blood metabolome, gut microbiota, testicular metabolome

## Abstract

High-fat diet (HFD)-induced obesity is known to be associated with reduced male fertility and decreased semen quality in humans. HFD-related male infertility is a growing issue worldwide, and it is crucial to overcome this problem to ameliorate the distress of infertile couples. For the first time, we discovered that fecal microbiota transplantation (FMT) of alginate oligosaccharide (AOS)-improved gut microbiota (A10-FMT) ameliorated HFD-decreased semen quality (sperm concentration: 286.1 ± 14.1 versus 217.9 ± 17.4 million/mL; sperm motility: 40.1 ± 0.7% versus 29.0 ± 0.9%), and male fertility (pregnancy rate: 87.4 ± 1.1% versus 70.2 ± 6.1%) by benefiting blood and testicular metabolome. A10-FMT improved HFD-disturbed gut microbiota by increasing gut *Bacteroides* (colon: 24.9 ± 1.1% versus 8.3 ± 0.6%; cecum: 10.2 ± 0.7% versus 3.6 ± 0.7%) and decreasing *Mucispirillum* (colon: 0.3 ± 0.1% versus 2.8 ± 0.4%; cecum: 2.3 ± 0.5% versus 6.6 ± 0.7%). A10-FMT benefited gut microbiota to improve liver function by adjusting lipid metabolism to produce n-3 polyunsaturated fatty acids, such as eicosapentaenoic acid (blood: 55.5 ± 18.7 versus 20.3 ± 2.4) and docosahexaenoic acid (testis: 121.2 ± 6.2 versus 89.4 ± 6.7), thus ameliorating HFD-impaired testicular microenvironment to rescue spermatogenesis and increase semen quality and fertility. The findings indicated that AOS-improved gut microbiota may be a promising strategy to treat obesity or metabolic issues-related male infertility in the future.

**IMPORTANCE** HFD decreases male fertility via upsetting gut microbiota and transplantation of AOS-benefited gut microbiota (A10-FMT) improves gut microbiota to ameliorate HFD-reduced male fertility. Moreover, A10-FMT improved liver function to benefit the blood metabolome and simultaneously ameliorated the testicular microenvironment to turn the spermatogenesis process on. We demonstrated that AOS-benefited gut microbiota could be applied to treat infertile males with obesity and metabolic issues induced by HFD.

## INTRODUCTION

Male infertility is a relatively common issue that is distressing for many couples of reproductive ages (1–4). Obesity is known to be associated with reduced male fertility and decreased semen quality in humans (5–9). HFD is a known risk factor for inducing obesity in animals and humans (6, 10). It has been reported that HFD decreases semen quality by lowering sperm concentration, reducing sperm motility, and increasing sperm abnormalities (1, 11). Recently, our group and others have found that gut dysbiosis (by HFD, anticancer drugs, or others) impairs spermatogenesis to diminish semen quality and/or male fertility (1, 11–13). Moreover, FMT was found to be an effective approach for ameliorating semen quality (12, 13).

Because obesity-related male infertility is a widespread global issue and HFD is one of its main causes, it is crucial to overcome this problem to ameliorate the distress of infertile couples (9, 14, 15). However, very few approaches have been successful. It is not understood whether gut microbiota could effectively ameliorate HFD decreased semen quality. Because dysbiosis of gut microbiota causes a reduction in semen quality, we proposed that recovering (or improving) the gut microbiota may improve or rescue semen quality or even restore male fertility. Therefore, we aimed to explore the beneficial improvement of AOS-modified gut microbiota on HFD-diminished semen quality and male fertility because it is effective in ameliorating semen quality in busulfan-treated subjects (12). Indeed, beneficial microbiota from AOS-dosed animals was demonstrated to significantly improve HFD induced decreases in sperm quality and male fertility.

## RESULTS

### A10-FMT improves HFD-diminished semen quality and male fertility.

**FIG 1:**
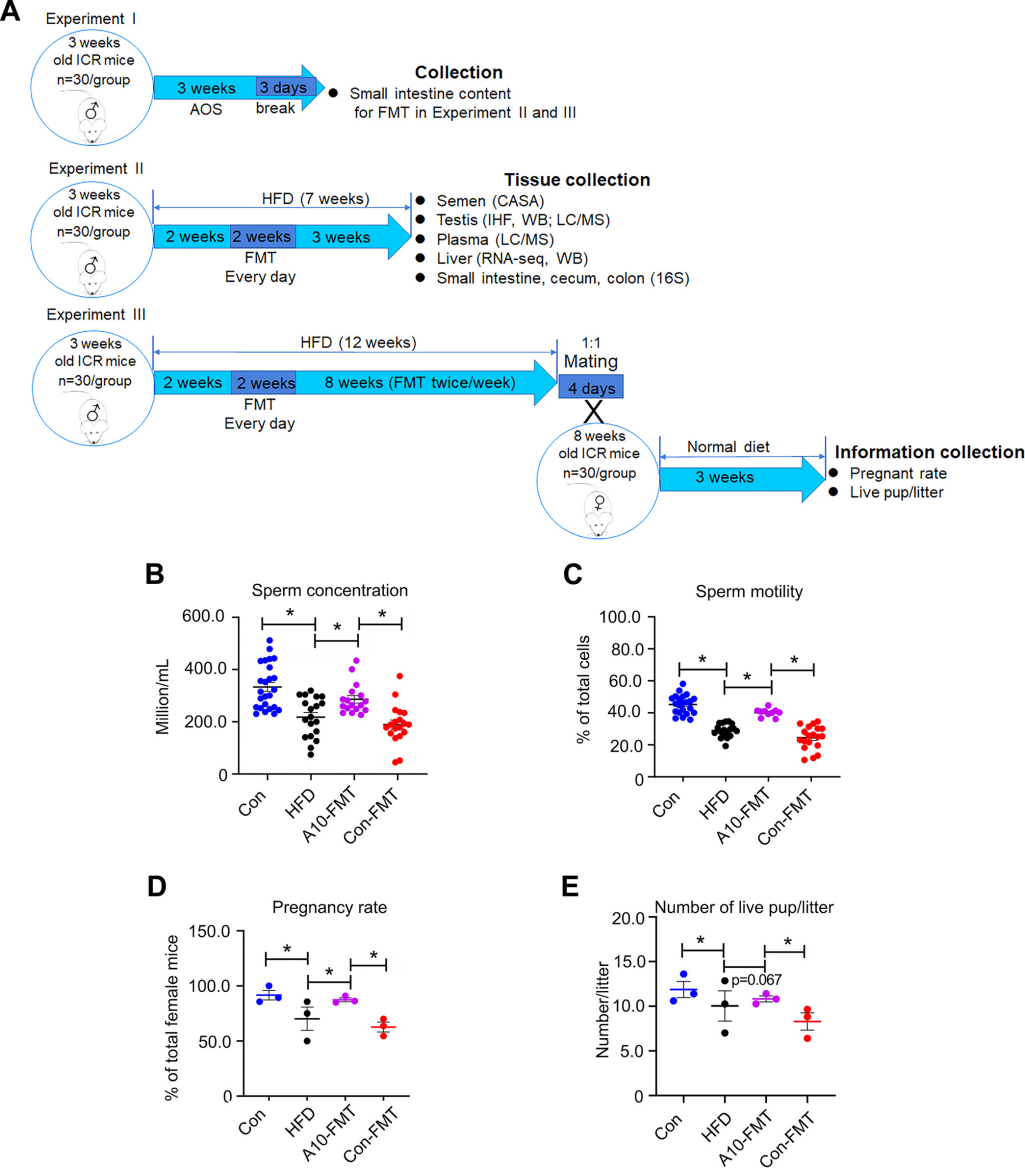
A10-FMT improved HFD-diminished semen quality and male fertility, and gut microbiota. (A) Experimental design. (B) Sperm concentration. The y-axis represents the concentration. The x-axis represents the treatment (*n* = 30/group). (C) Sperm motility. The y-axis represents the percentage of cells. The *x*-axis represents the treatment (*n* = 30/group). (D) Pregnancy rate (number of pregnant animals/total animals). (E) The average number of live pups/litter. *, *P* < 0.05.

In the current study, HFD significantly increased body weight compared to the control group, while fecal microbiota transplantation (FMT) from AOS dosed animals (A10-FMT) and control animals (Con-FMT) caused a decrease, compared to HFD (Fig. S1A). Consistent with other reports (1, 11), HFD decreased sperm concentration and sperm motility (Fig. 1B and C). A10-FMT significantly increased sperm concentration (to levels similar to the control) and sperm motility compared to that of HFD, while Con-FMT did not (Fig. 1B and C). Interestingly, A10-FMT rescued HFD-decreased male fertility through an elevation in the pregnancy rate and the number of offspring per litter; however, Con-FMT did not change these parameters (Fig. 1D and E). Data from the current investigation and our earlier reports (12, 13) confirm that A10-FMT does indeed improve spermatogenesis and male fertility.

### A10-FMT improves HFD-upset gut microbiota.

**FIG 2:**
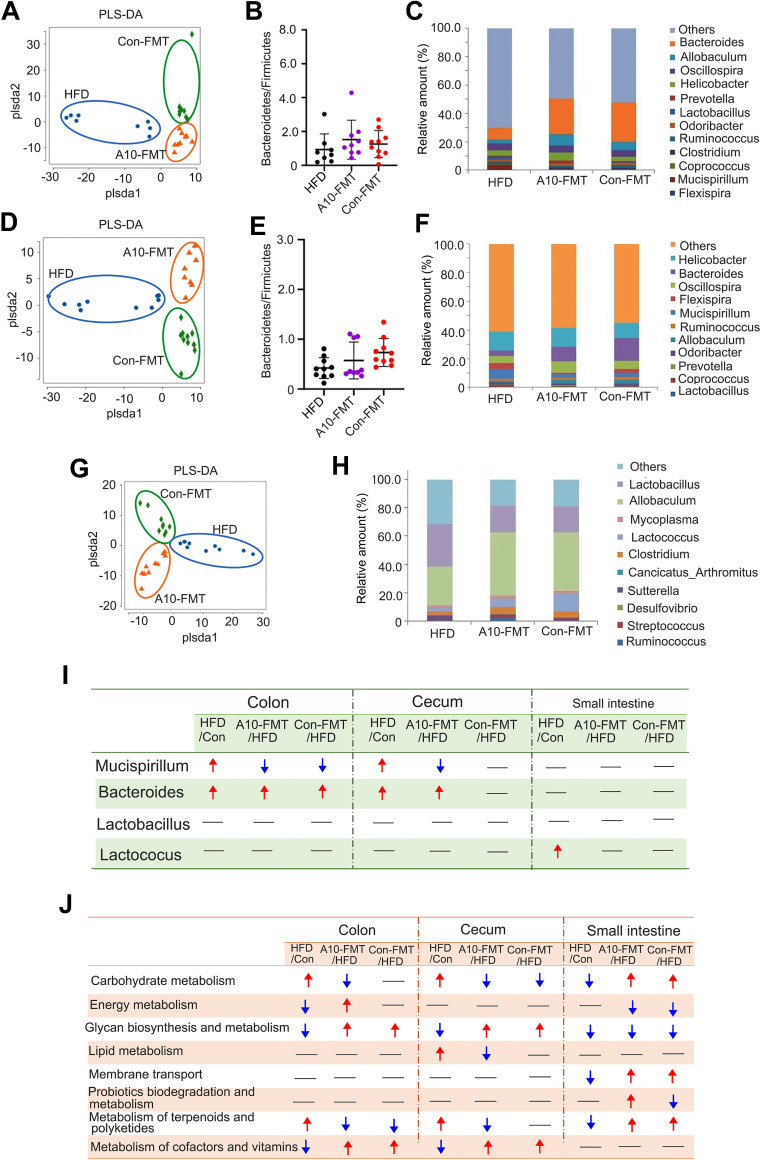
Gut microbiota changes among HFD, A10-FMT, and Con-FMT. (A) PLS-DA (OTU) of colon microbiota in HFD, A10-FMT, and Con-FMT groups. (B) The ratio of Bacteroidetes/Firmicutes in the colon in HFD, A10-FMT, and Con-FMT groups. (C) Colon microbiota levels at genus level in HFD, A10-FMT, and Con-FMT groups. The y-axis represents the relative amount (%). The x-axis represents the treatments. Different color represents different microbiota. (D) PLS-DA (OTU) of cecum microbiota in HFD, A10-FMT, and Con-FMT groups. (E) The ratio of Bacteroidetes/Firmicutes in cecum in HFD, A10-FMT, and Con-FMT groups. (F) Cecum microbiota levels at genus level in HFD, A10-FMT, and Con-FMT groups. The y-axis represents the relative amount (%). The x-axis represents the treatments. Different color represents different microbiota. (G) PLS-DA (OTU) of small intestine microbiota in HFD, A10-FMT, and Con-FMT groups. (H) Small intestine microbiota levels at genus level in HFD, A10-FMT, and Con-FMT groups. The y-axis represents the relative amount (%). The x-axis represents the treatments. Different color represents different microbiota. (I) Summary of the changed gut microbiota in colon, cecum, and small intestine in different treatments. The red arrow indicates increased microbiota in each comparison. The blue arrow indicates decreased microbiota in each comparation. (J) Summary of signaling pathways of changed microbiota genes by KEGG enrichment analysis. The red arrow indicates increased microbiota genes in each comparison. The blue arrow indicates decreased microbiota genes in each comparison.

To explore how A10-FMT improves HFD-diminished male fertility, we investigated gut microbiota modification after HFD and/or A10-FMT treatment. In the current study, we separately explored the gut microbiota in the small intestine, cecum, and colon. Accordingly, the partial least squares discriminant analysis (PLS-DA) (at operational taxonomic unit [OTU]) clearly showed differences in bacterial composition between the HFD and Con-FMT groups (Fig. S1B, D, and F), and among HFD, A10-FMT (HFD +A10-FMT), and Con-FMT (HFD +Con-FMT) (Fig. 2A, D, and G). A10-FMT and Con-FMT increased the ratio of Bacteroidstes/Firmicutes in the cecum and colon (1, 12), which indicated that these two treatments benefited gut microbiota (Fig. 2B and E). At the genus level, the main changed gut microbiotas were *Mucispirillum* and *Bacteroides* (Fig. 2C, F, H, and I; Fig. S1C, E, and G). Compared to Con-FMT, HFD increased the amount of these two microbiotas in the colon and cecum but not in the small intestine (Fig. 2I). A10-FMT decreased *Mucispirillum* in the colon and cecum, while Con-FMT reduced *Mucispirillum* in the colon but not in the cecum (Fig. 2I). On the other hand, A10-FMT increased *Bacteroides* in the colon and cecum, while Con-FMT elevated *Bacteroides* in the colon but not in the cecum (Fig. 2I). In addition, compared to Con-FMT, HFD significantly increased the amount of *Lactococcus* in the small intestine. However, A10-FMT and Con-FMT did not change this microbiota (Fig. 2I).

The gut microbiota participates in host metabolism by interacting with host signaling pathways (16). Kyoto Encyclopedia of Genes and Genomes (KEGG) analysis of changed microbiota genes indicated that eight major signaling pathways were upset by HFD and recovered by A10-FMT and/or Con-FMT in the colon, cecum, and/or small intestine (Fig. 2J). Interestingly, the “lipid metabolism” pathway was upset by HFD while reversed by A10-FMT, and “probiotic biodegradation and metabolism” was changed in the opposite way by A10-FMT and Con-FMT (Fig. 2J). It is known that HFD upsets lipid metabolism because the host and gut microbiota interact together to support this process (16). Furthermore, it has been reported that the gut microbiota *Mucispirillum* is involved in lipid metabolism (2, 12), and it was increased by HFD while decreased by A10-FMT.

### A10-FMT-benefited gut microbiota improves liver lipid metabolism through bile acid-retinoic acid pathways to ameliorate HFD disrupted blood metabolome.

**FIG 3:**
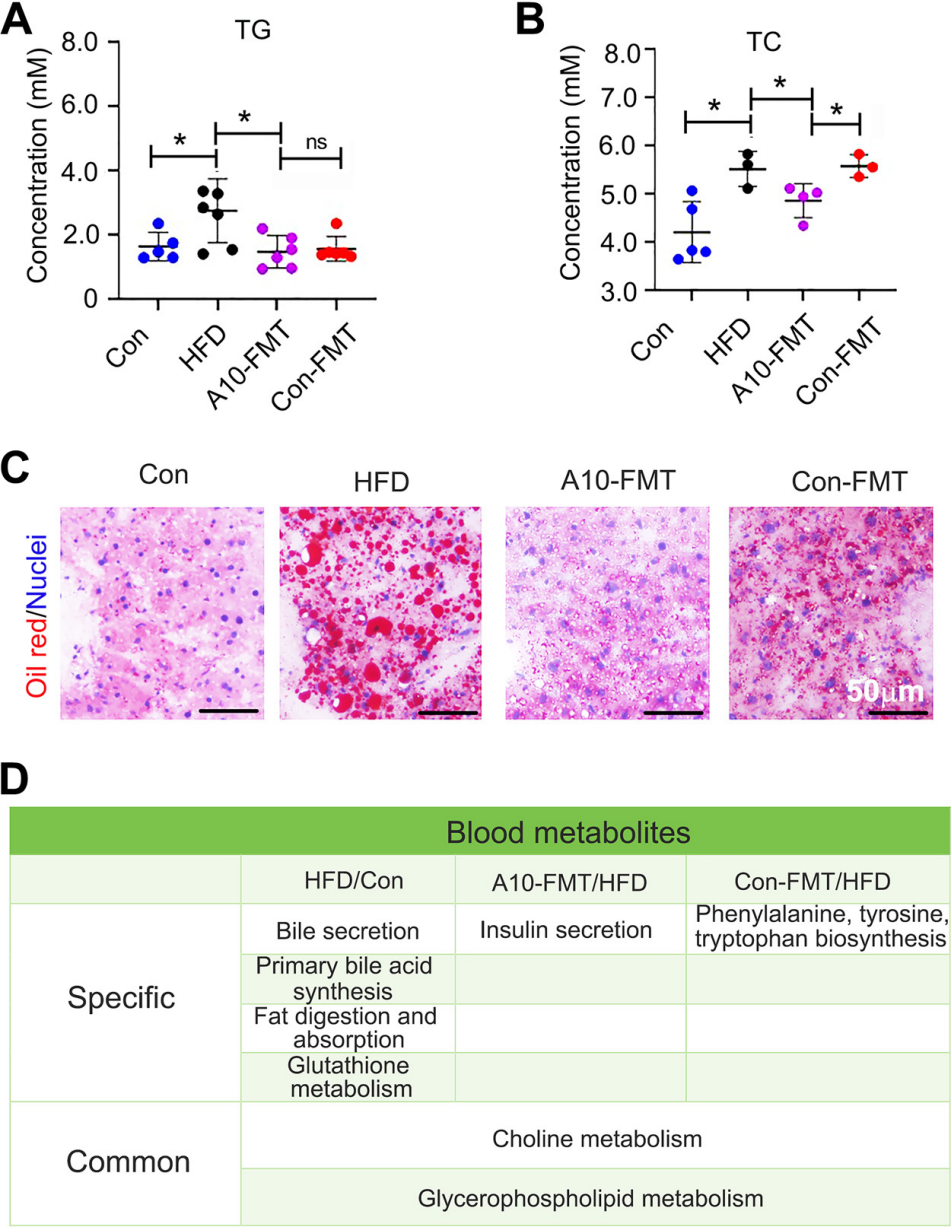
A10-FMT benefited blood metabolism and liver lipid metabolism. (A) Blood total glycerides (TG) levels changed by HFD, A10-FMT, and Con-FMT. The y-axis represents the concentration. The x-axis represents the treatment. *, *P* < 0.05. (B) Blood total cholesterol (TC) levels changed by HFD, A10-FMT, and Con-FMT. The y-axis represents the concentration. The x-axis represents the treatment. *, *P* < 0.05. (C) Liver lipid status by oil red staining. Scale bar: 50 mm. (D) Summary of functional pathways of changed blood metabolites by KEGG enrichment analysis. The blood metabolites were determined by HPLC quantification.

It is known that HFD induces obesity and increases blood lipid levels. Next, we explored the blood lipid status by studying total glycerides (TG) and total cholesterol (TC). Compared to the control group, HFD increased blood TG and TC levels, while A10-FMT and Con-FMT reversed TG to the level found in the control group (Fig. 3A and B). A10-FMT reduced blood TC to control group levels, but Con-FMT did not (Fig. 3B). The liver is the major organ for lipid synthesis and metabolism (15). HFD dramatically increased liver lipids, while A10-FMT showed a greater impact on reducing liver lipid content than Con-FMT (Fig. 3C; Fig. S2A). Blood metabolism analysis showed that there were distinct differences between the HFD and the control groups, A10-FMT and HFD or Con-FMT and HFD (Fig. S3A to C; Data Set S2). KEGG functional enrichment analysis of changed blood metabolites showed that HFD did significantly change blood bile acids via two functional pathways “bile secretion” and “primary bile acid synthesis” that were found in the HFD/Con (Fig. 3D). Another important functional pathway “fat digestion and absorption” was present for the HFD/Con (Fig. 3D). The functional pathway “insulin secretion” was only enriched in A10-FMT/HFD, and “phenylalanine, tyrosine, tryptophan biosynthesis” was only enriched in Con-FMT/HFD (Fig. 3D). “Choline metabolism” and “glycerophospholipid metabolism” were found in all three comparisons: HFD/Con, A10-FMT/HFD, and Com-FMT/HFD.

**FIG 4:**
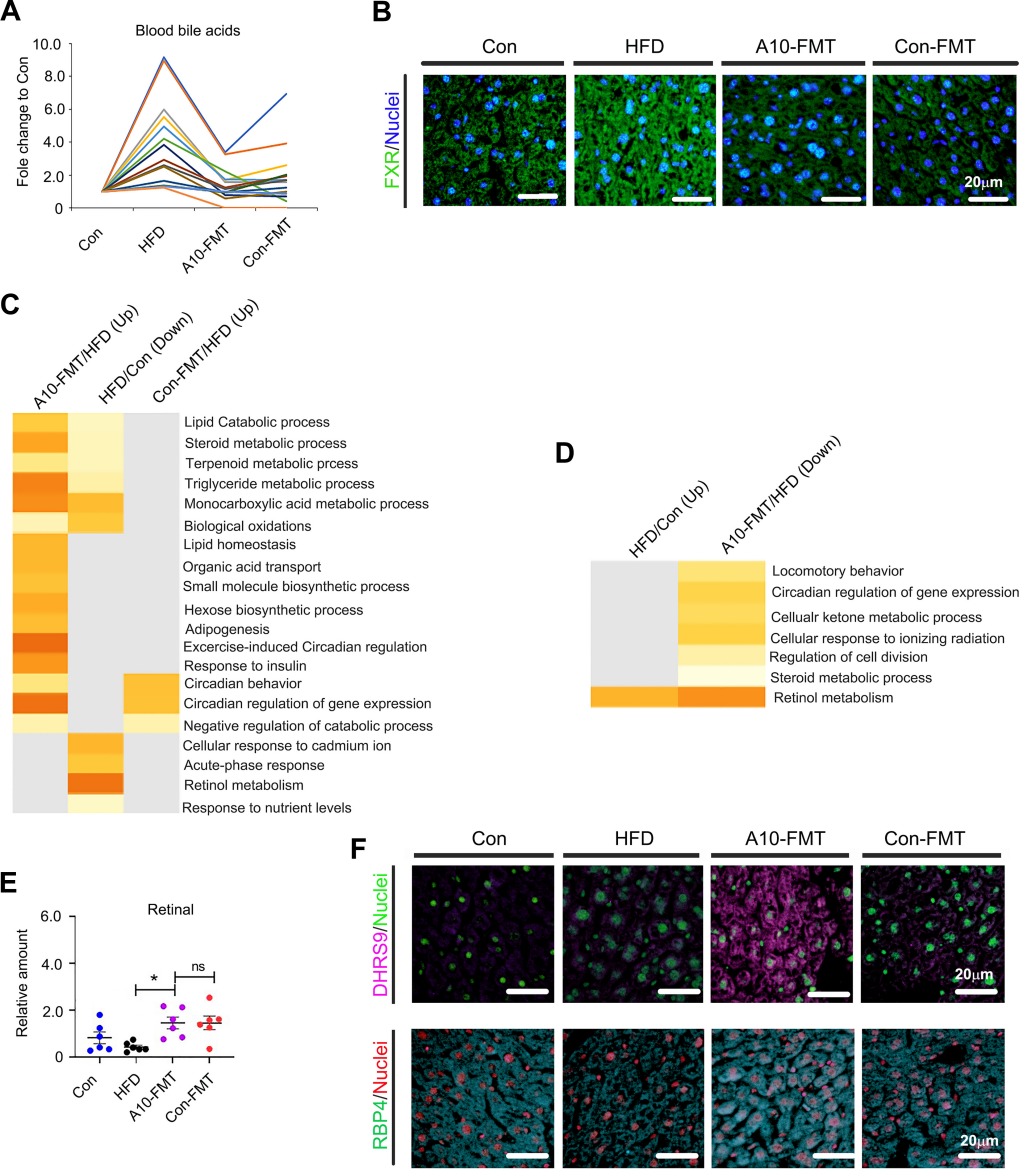
A10-FMT ameliorated liver bile acid and retinol metabolism. (A) Summary of the blood changed bile acids (by HPLC quantification) in each treatment. (B) IHF staining of liver bile acid receptor FXR. Scale bar: 20 mm. (C) Gene functional enrichment analysis of genes decreased by HFD while increased by A10-FMT or Con-FMT of the RNA-seq analysis of liver tissue by online Metascape software (https://metascape.org/gp/index.html#/main/step1). (D) Gene functional enrichment analysis of genes increased by HFD while decreased by A10-FMT of the RNA-seq analysis of liver tissue by online Metascape software. (E) Blood retinal levels (by HPLC quantification) in each treatment. The y-axis represents the concentration. The x-axis represents the treatment. *, *P* < 0.05. (F) Immunofluorescence (IHF) staining of liver retinol synthesis protein DHRS9 and retinol-binding protein RBP4 in each treatment. Scale bar: 20 mm.

Moreover, we found that HFD increased blood bile acids levels while A10-FMT decreased them (Fig. 4A). All these results suggested that the liver bile acid signaling pathway would change and that HFD increased the protein levels of the bile acid receptor FXR, which was decreased by A10-FMT and Con-FMT (Fig. 4B). Bile acids play vital roles in intestinal nutrient absorption and biliary secretion of lipids (17–19). Moreover, bile acids and the retinoic acid (RA) signaling pathway interact together to regulate lipid metabolism (20–22). The liver RNA-seq analysis showed that lipid metabolism-related signaling functional pathways were enriched for genes decreased by HFD while increased by A10-FMT (Fig. 4C; Fig. S2B). Moreover, the “retinol metabolism” signaling pathway was enriched for those genes increased by HFD while decreased by A10-FMT (Fig. 4D). Blood retinal level was decreased by HFD, while it was significantly increased by A10-FMT (Fig. 4E). Retinol and RA were decreased by HFD and increased by A10-FMT in the liver (Fig. S2C and D). Furthermore, the retinol synthesis enzyme DHRS9 was increased by A10-FMT (Fig. 4F), and the retinol-binding protein RPB4 (important for retinol storage) was decreased by HFD and increased by A10-FMT in the liver (Fig. 4F). Moreover, fatty acid levels in the blood were upset by HFD and reversed by A10-FMT (Fig. S3D to F).

**FIG 5:**
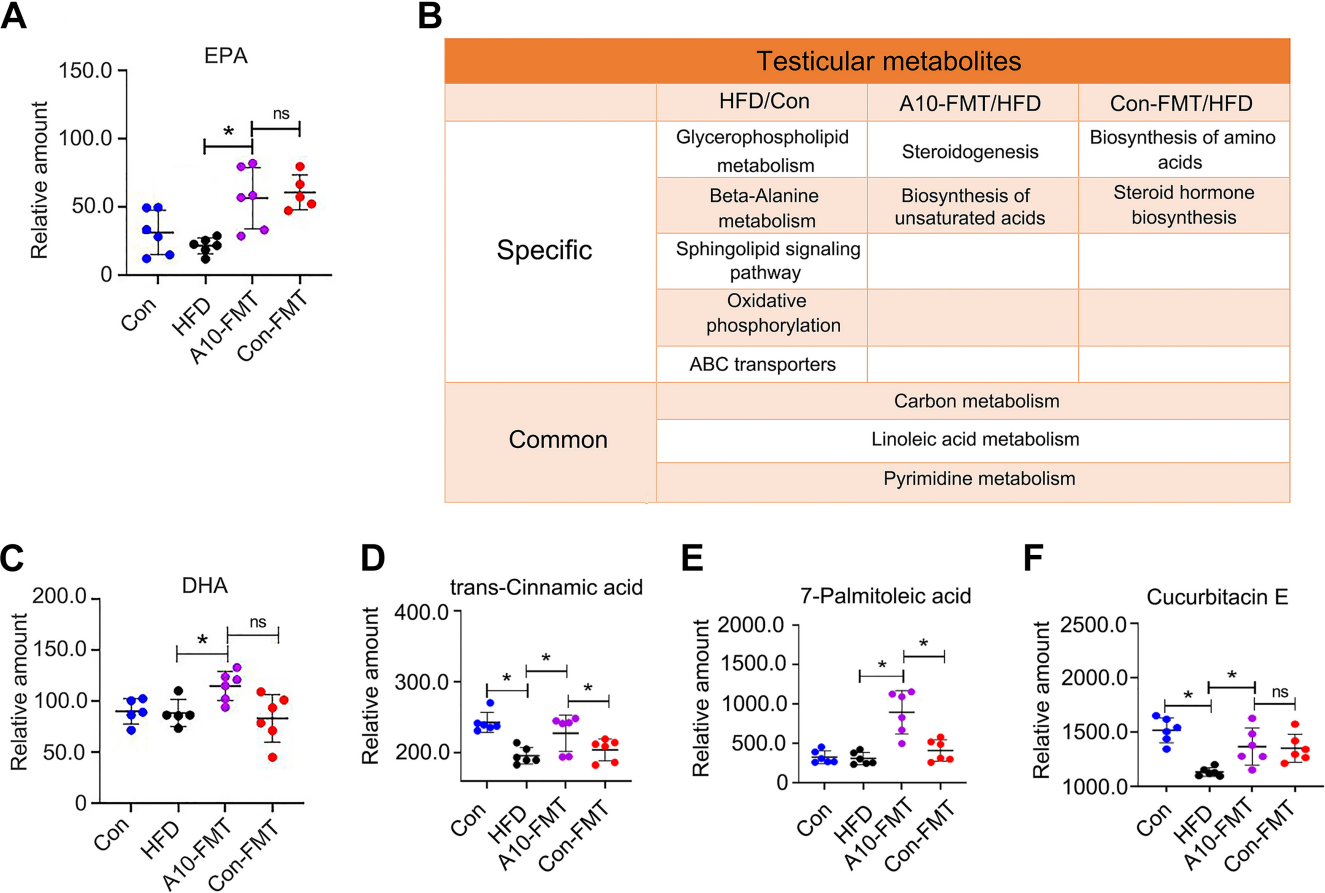
A10-FMT improved blood and testicular PUFA. (A) Blood EPA levels (by HPLC quantification) in each treatment. The y-axis represents the concentration. The *x*-axis represents the treatment. *, *P* < 0.05. ns means not significant. (B) Summary of functional pathways of changed testicular metabolites by KEGG enrichment analysis. The testicular metabolites were determined by HPLC quantification. (C) Testicular DHA levels (by HPLC quantification) in each treatment. The y-axis represents the concentration. The *x*-axis represents the treatment. *, *P* < 0.05. ns means not significant. (D) Testicular trans-cinnamic acid levels (by HPLC quantification) in each treatment. The y-axis represents the concentration. The *x*-axis represents the treatment. *, *P* < 0.05. (E) Testicular 7-Palmitoleic acid levels (by HPLC quantification) in each treatment. The y-axis represents the concentration. The x-axis represents the treatment. *, *P* < 0.05. (F) Testicular Cucurbitacin E levels (by HPLC quantification) in each treatment. The y-axis represents the concentration. The x-axis represents the treatment. *, *P* < 0.05.

Most interesting was that the blood n-3 polyunsaturated fatty acid eicosapentaenoic acid (EPA) was increased by A10-FMT (Fig. 5A). It is known that RA regulates the expression of fatty acid desaturases such as stearoyl-CoA desaturase and delta-5 desaturase, to control polyunsaturated fatty acids (PUFA) levels (22). All the data confirmed that bile acids and RA signaling pathways were involved in A10-FMT improving lipid metabolism.

### A10-FMT-improved blood metabolites ameliorate testicular metabolome (microenvironment) to rescue HFD disrupted spermatogenesis.

**FIG 6:**
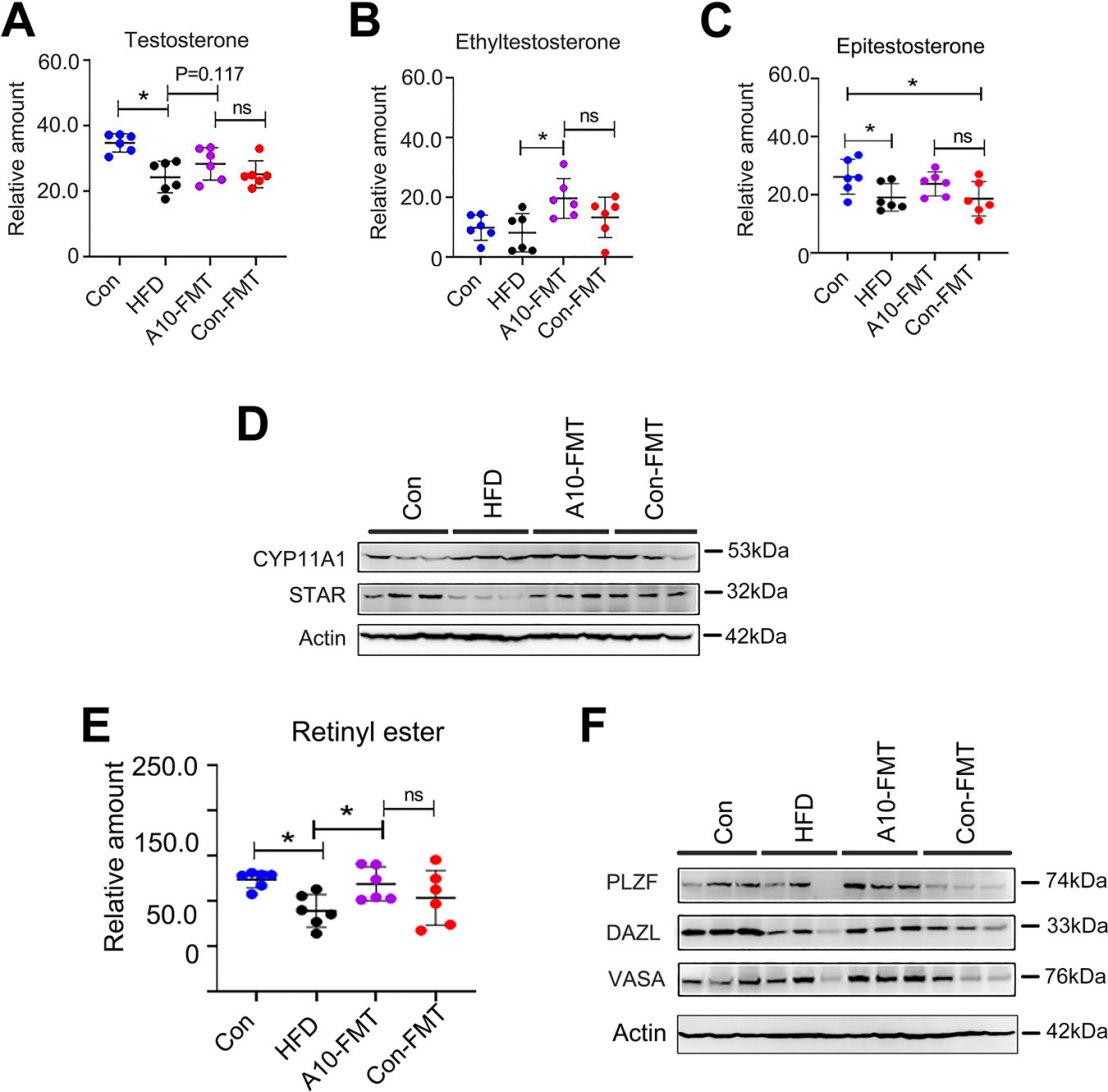
A10-FMT benefited testicular testosterone levels and synthesis, and retinol levels to initiate spermatogenesis. (A) Testicular testosterone levels (by HPLC quantification) in each treatment. The y-axis represents the concentration. The x-axis represents the treatment. *, *P* < 0.05. ns, not significant. (B) Testicular ethyltestosterone levels (by HPLC quantification) in each treatment. The y-axis represents the concentration. The x-axis represents the treatment. *, *P* < 0.05. ns means not significant. (C) Testicular Epitestosterone levels (by HPLC quantification) in each treatment. The y-axis represents the concentration. The x-axis represents the treatment. *, *P* < 0.05. (D) Western blotting of testicular important proteins for steroid hormone production in each treatment. (E) Testicular Retinyl ester levels (by HPLC quantification) in each treatment. The *y-*axis represents the concentration. The *x*-axis represents the treatment. *, *P* < 0.05. (F) Western blotting of testicular important proteins for spermatogenesis onset.

**FIG 7:**
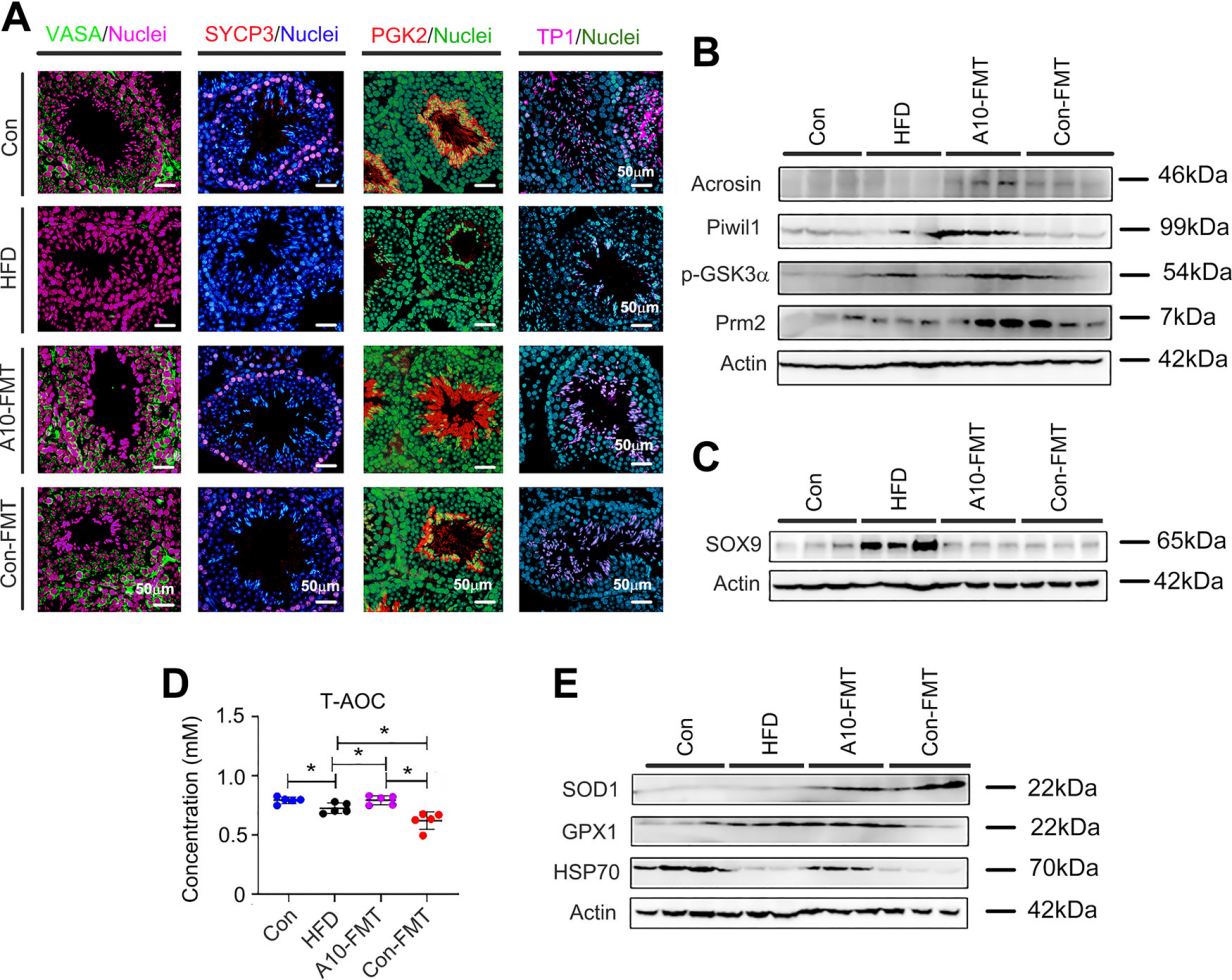
A10-FMT improved the spermatogenesis process and antioxidant capability. (A) IHF staining of testicular germ cell marker VASA, meiosis marker SYCP3, sperm protein PGK2, and transition protein 1 (TP1) in each treatment. Scale bar: 50 mm. (B) Western blotting of testicular important proteins for spermatogenesis in each treatment. (C) Western blotting of Sertoli cell marker SOX9 in each treatment. (D) Blood T-AOC levels in each treatment. The y-axis represents the concentration. The x-axis represents the treatment. *, *P* < 0.05. (E) Western blotting of testicular antioxidant-related proteins in each treatment.

It is well known that lipids/fatty acids especially polyunsaturated fatty acids (PUFA) play important roles in spermatogenesis; in particular, docosahexaenoic acid (DHA) is crucial (23, 24). The functional enrichment of testicular metabolites showed that the functional pathway “steroidogenesis” and “biosynthesis of unsaturated acids” were enriched in A10-FMT/HFD (Fig. 5B; Fig. S4A to C; Data Set S3). Especially, A10-FMT increased testicular unsaturated acids, such as DHA (Fig. 5C to F; Fig. S4D). Moreover, testicular testosterone levels were elevated by A10-FMT (Fig. 6A to C; Fig. S4E). The protein levels of steroid hormone synthesis enzymes CYP11A1 and StAR were decreased by HFD and increased by A10-FMT (Fig. 6D), which confirmed that testicular testosterone was increased by A10-FMT (Fig. 6A to C). Furthermore, retinol and RA are vital for germ cell development and the meiosis process during spermatogenesis (25, 26), and at the same time, PUFA can bind to RA receptors to activate RA signaling (27, 28). Here, A10-FMT increased the testicular level of retinol (Fig. 6E; Fig. S4F). Moreover, A10-FMT increased the testicular protein levels of spermatogonial cell makers DEAD-box helicase 4 (VASA), zinc finger And BTB domain containing 16 (PLZF), and deleted in azoospermia like (DAZL) (Fig. 6F), which indicated the spermatogenesis process turned on. All the data indicated that A10-FMT should improve the process of spermatogenesis, and this did take place (Fig. 7A and B). A10-FMT significantly increased the protein levels of meiosis marker SYCP3, transition protein TP1, sperm protein PGK2 (essential for sperm motility and male fertility), and Acrosin, Piwil1, p-GSK3a, and Prm2 (1,2), while these were all decreased by HFD (Fig. 7A and B). However, Con-FMT did increase these proteins slightly (Fig. 7A and B). Furthermore, the Sertoli cell marker SOX9 (1,2) was increased by HFD and reversed by A10-FMT (Fig. 7C).

Additionally, antioxidant capacity in the blood and testes was improved by A10-FMT (Fig. 7D and E). Blood T-AOC was decreased by HFD while increased by A10-FMT, not by Con-FMT. The testicular protein levels of SOD1, GPX1, and HSP70 were lower in the HFD group while were elevated by A10-FMT. The data suggest that the increased antioxidant capability may also have a contribution to A10-FMT improved spermatogenesis.

## DISCUSSION

Infertility, a global health issue, is reported to influence more than 180 million people worldwide (~30% of reproductive‐aged couples) (1, 2, 10). Infertility in women has historically been the focus of attention; however, the male factor is involved in up to 70% of infertility cases owing to inadequate production of motile and functional sperm (10). In the last 40 years, sperm counts and motility have continued to decrease by over 50% in Western countries and there are no signs that this adverse trend is slowing (3, 10). Equally concerning are findings that high proportions of young men have sperm concentrations falling within the subfertile range (10). It is unknown why this rapid decline in semen quality has taken place but there appears to be an environmental rather than genetic cause (2, 10). Several environmental factors are likely contributing to the decline in semen quality: environmental toxins, pesticides, heat, stress, smoking, alcohol consumption, and the use of mobile phones and wireless Internet (2, 10, 12). These multiple factors may have cumulative and interacting effects leading to a reduction in semen quality. However, one environmental factor that is outstanding is dietary alteration and the consequent increase in body weight, and it has shown high rates of change over recent decades concurrent with declining rates of semen quality (10, 29, 30). “Westernized” dietary changes have long been considered an important contributing factor to the protracted global decline of sperm concentrations in humans (1). Although many studies propose the need to overcome this adverse impact, there has been little progress (9, 14). Ding et al. (1) found that HFD disrupts the gut microbiota to decrease semen quality. This suggests that male infertility could be addressed by restoring gut microbial ecosystems. Indeed, herein we found that an improvement in gut microbiota reversed HFD-impaired spermatogenesis, to increase semen quality and male fertility. Moreover, we have shown similar results in two study models (HFD model and busulfan model) (12, 13), demonstrating that AOS-benefited gut microbiota (A10-FMT) (31) has a strong potential to increase semen quality and male fertility. In the busulfan model, A10-FMT benefited gut microbiota by increasing the proportions of *Bacillales*, *Bacteroidales*, and *Bifidobacteriales*, while in the HFD study, A10-FMT elevated *Bacteroidales* and decreased *Mucispirillum* were reported to correlate with lipid metabolism (32, 33). The data indicated that AOS-benefited gut microbiota (A10-FMT) can improve gut microbiota under different conditions. Moreover, Qian et al. (34) reported that chitosan oligosaccharide decreased mouse blood triglycerides (TGs) and free fatty acids (FFAs) with the reduction of the abundance of *Mucispirillum* in gut microbiota (2019). It has been reported that high-fat diets contribute to the onset of obesity (22, 35). Similarly, we found that HFD increased animal body weight while A10-FMT and Con-FMT decreased body weight. This confirmed that gut microbiota (A10-FMT) contributes to lipid metabolism (22).

Bile acids and RAs are crucial for liver lipid metabolism (36, 37). RA receptor (retinoid x receptor) and bile acid receptor (farnesoid x receptor, FXR/NR1H4) act as partners (37). In addition, RA and bile acid interact together in regulating lipid homeostasis and insulin sensitivity (20, 21). A10-FMT improved the expression of bile acid receptors and the proteins for retinol synthesis and storage. The liver and blood lipid status has been improved by A10-FMT, which was consistent with another report (38). Furthermore, RA can regulate fatty acid desaturation to produce PUFA (22). Blood essential n-3 PUFA, EPA was increased by A10-FMT, at the same time testicular n-3 PUFA, DHA was elevated by A10-FMT in the current investigation. The data suggest that A10-FMT improved PUFA levels which contributed to spermatogenesis (24, 39). In the testes, PUFA and RA interact together to regulate spermatogenesis. PUFA such as DHA binds to RXRα to modify RA actions (27, 28). On the other hand, retinol and RA control male germ cell fate, the meiosis process, and development during spermatogenesis (25, 26, 40). A10-FMT increased testicular levels of retinal, DHA that interacted together to initiate the spermatogenesis process by the increase in the testosterone levels to increase the expression of proteins important for spermatogenesis.

In summary, we presented transplantation of AOS-benefited gut microbiota (A10-FMT) improved gut microbiota to ameliorate liver function through alterations in lipid metabolism by the interaction of bile acid and RA, then the blood metabolome was improved with the increase in the n-3 PUFA, EPA. Simultaneously, testicular retinal and DHA levels were improved by A10-FMT to turn the spermatogenesis process on. HFD impaired semen quality and male fertility were then rescued. We demonstrate that AOS-benefited gut microbiota might be applied to treat infertile males with obesity and metabolic issues, however, human studies should be done to validate the findings before applied to the clinic.

## MATERIALS AND METHODS

### Study design and ethics.

All animal procedures used in this study were approved by the Animal Care and Use Committee of the Institute of Animal Sciences of the Chinese Academy of Agricultural Sciences (IAS2020-106). Mice were maintained in a specific pathogen-free (SPF) environment under light:dark cycle of 12:12 h, at a temperature of 23°C and humidity of 50%–70%; they had free access to food (chow diet) and water (12, 13, 31).

**Experiment I.** Mouse small intestine microbiota collection (12, 13). 3-week-old ICR male mice were dosed with ddH_2_O as the control or AOS 10 mg/kg BW via oral gavage (0.1 mL/mouse/d). AOS dosing solution was freshly prepared daily and delivered every morning for 3 weeks. There were two groups (30 mice/treatment): (i) control (ddH_2_O) and (ii) A10 (AOS 10 mg/kg BW). After 3 weeks of treatment, the animals were maintained on a regular diet for three more days (no treatment). The mice were then humanely euthanized to collect small intestinal luminal content (microbiota) (Fig. 1A).

**Experiment II.** HFD feeding and fecal microbiota transplantation (FMT; short-term experiment [7 weeks HFD feeding]) (1, 12, 13, 41–43). The small intestine luminal content (microbiota) from each group was pooled and homogenized, diluted 1:1 in 20% sterile glycerol (saline), and frozen. Before inoculation, fecal samples were diluted in sterile saline to a working concentration of 0.05 g/mL and filtered through a 70-μm cell strainer. Three-week-old ICR male mice were used in the current investigation. There were four treatment groups (30 mice/treatment): (i) control (regular diet plus Saline); (ii) HFD (an HFD [Research Diet D12492, 60% fat] (1) plus saline); (iii) Con-FMT (HFD plus gut microbiota from control mice [Experiment I]); (iv) A10-FMT (HFD plus gut microbiota from AOS 10 mg/kg mice [Experiment I]). Mice were fed with HFD or control diet for 2 weeks (3 weeks of age to 5 weeks of age) then the mice received oral FMT inoculations (0.1 mL) once daily for 2 weeks (5 weeks of age to 7 weeks of age). The mice were then regularly maintained (on their respective diet) for another 3 weeks (10 weeks of age). The mice were humanely euthanized to collect samples for different analyses (Fig. 1A).

**Experiment III.** HFD feeding, microbiota transplantation (FMT), and fertility experiment (long term experiment [12 weeks HFD feeding]) (1, 12). This experiment was similar to Experiment II. There were four treatment groups (30 mice/treatment): (i) control (regular diet plus saline); (ii) HFD (an HFD [Research Diet D12492, 60% fat] plus Saline); (ii) Con-FMT (HFD plus gut microbiota from control mice [Experiment I]); (iv) A10-FMT (HFD plus gut microbiota from AOS 10 mg/kg mice [Experiment I]). Mice were fed with HFD or control diet for 2 weeks (3 weeks of age to 5 weeks of age) then the mice received oral FMT inoculations (0.1 mL) once daily for 2 weeks (5 weeks of age to 7 weeks of age). The mice received FMT inoculations twice per week (Monday and Thursday) for 8 weeks. The mice were maintained on their respective diet for the whole 12 weeks (15 weeks of age). The mice were mated with regular ICR female mice (male 1:1 female) for 4 days (12). The male mice were then taken out and humanely euthanized to collect samples for different analyses. The female mice were maintained on a regular diet (control diet). The pregnancy rate and the number of live pups/litter were determined (Fig. 1A).

### Evaluation of spermatozoa motility using a computer-assisted sperm analysis system.

Spermatozoa motility was assessed using a computer-assisted sperm assay (CASA) method according to World Health Organization guidelines and reported in our early article (31).

### Morphological observations of spermatozoa.

The extracted murine caudal epididymides were placed in Roswell Park Memorial Institute (RPMI) 1640 medium, finely chopped, and then Eosin Y (1%) was added for staining as described previously (31).

### Assessment of acrosome integrity.

The integrity of acrosome was assessed as in our early report (31). Briefly, the sperm suspension was uniformly smeared on a clean glass slide, air-dried, and incubated in methanol for 2 min for fixation. After fixation, the slides with the sperm were stained with 0.025% Coomassie brilliant blue G-250 in 40% methanol for 20 min at room temperature (RT). Acrosomal integrity was determined by intense staining on the anterior region of the sperm head under bright-field microscopy (AH3-RFCA, Olympus, Tokyo, Japan) and scored accordingly (31).

### RNA Isolation and RNA-seq analyses.

Briefly, total RNA was isolated using TRIzol Reagent (Invitrogen) and purified using a Pure-Link1 RNA Minikit (catalog number 12183018A; Life Technologies) following the manufacturer's protocol. The sequencing and data analysis were reported in our early article (31). Briefly, the library products were prepared for sequencing in an Illumina HiSeqTM 2500. The reads were mapped to reference genes using SOAPaligner (v. 2.20) with a maximum of two nucleotide mismatches allowed at the parameters of “-m 0 -x 1000 -s 40 -l 35 -v 3 -r 2”. The read number of each gene was transformed into RPKM (reads per kilobases per million reads), and then differentially expressed genes were identified using the DEGseq package and the MARS (MA-plot-based method with random sampling model) method. The threshold was set as false discovery rate (FDR) ≤0.001 and an absolute value of log_2_ ratio ≥1 to judge the significance of the difference in gene expression. The data were then analyzed by GO enrichment and KEGG enrichment.

### Sequencing of microbiota from intestine digesta samples and data analysis.

The sequencing and analysis of gut microbiota were reported in our early study (31). Briefly, the total genomic DNA of the small intestine, cecum, and colon digesta was isolated using an E.Z.N.A.R Stool DNA kit (Omega Bio-tek Inc., USA) following the manufacturer’s instructions. DNA quantity and quality were analyzed using NanoDrop 2000 (Thermo Scientific, USA) and 1% agarose gel. Ten samples/groups were determined. The V3-V4 region of the 16S rRNA gene was amplified using the primers MPRK341F (5’-ACTCCTACGGGAGGCAGCAG-3′) and MPRK806R (5’-GGACTACHVGGGTWTCTAAT-3’) with barcodes. The sequencing libraries were constructed with NEB NextR UltraTM DNA Library Prep kit for Illumina (NEB, United States) following the manufacturer’s instructions and index codes were added. The library was sequenced on the Illumina HiSeq 2500 platform and 300 bp paired-end reads were generated at the Novo gene. Operational taxonomic unit abundance information was normalized using a standard sequence number corresponding to the sample with the least sequences. The alpha diversity index was calculated with QIIME (Version 1.7.0). The Unifrac distance was obtained using QIIME (Version 1.7.0), and PCoA (principal coordinate analysis) was performed using R software (Version 2.15.3). The linear discriminate analysis effect size (LEfSe) was performed to determine differences in abundance. The threshold linear discriminant analysis (LDA) score was 4.0. GraphPad Prism7 software was used to produce the graphs.

### Plasma and testis metabolite measurements by liquid chromatography-mass spectrometry (LC-MS/MS).

Plasma and testicular metabolites were determined as reported in our recent article (14, 31). Before LC-MS/MS analysis, the samples were thawed on ice and processed to remove proteins. Testis samples were collected and the same amount of tissue from each mouse testis was used to isolate the metabolites using CH3OH: H2O (V: V) = 4:1. The samples were then detected by ACQUITY UPLC and AB Sciex Triple TOF 5600 (LC/MS) as reported previously. Progenesis QI v2.3 (Nonlinear Dynamics, Newcastle, UK) was implemented to normalize the peaks. The Human Metabolome Database (HMDB), Lipidmaps (v2.3), and METLIN software were then used to qualify the data. Moreover, the data were processed with SIMCA software (version 14.0, Umetrics, Umeå, Sweden) following pathway enrichment analysis using the KEGG database (http://www.genome.jp/KEGG/pathway.html).

### Liver oil red staining.

The liver lipid formation was stained using an Oil Red O staining kit according to the manufacturer’s instructions (catalog number: D027; Nanjing Jiancheng Bioengineering Institute, Nanjing, People’s Republic of China) (43, 44).

### Determination of blood TG, TC, and T-AOC.

Blood TG, TC, and T-AOC were determined by the kits from Nanjing Jiancheng Bioengineering Institute (Nanjing, People’s Republic of China; TG [catalog number: A110-1-1]; TC [catalog number: A111-1-1]; T-AOC [catalog number: A015-2-1]) (44). All procedures followed the manufacturer’s instructions.

### Qualification of liver retinol and retinoic acids by high performance liquid chromatography (HPLC).

The liver content of retinol and retinoic acid were determined followed the reported methods by HPLC (45).

### Histopathological analysis.

Testicular tissues were fixed in 10% neutral buffered formalin, paraffin-embedded, cut into 5 μm sections, and subsequently stained with hematoxylin and eosin (H&E) for histopathological analysis.

### Western blotting.

Western blotting of proteins was carried out as previously reported (14, 15, 31). The information for primary antibodies is listed in Table S1.

### Detection of protein levels and location in testis using immunofluorescence staining.

The methodology for immunofluorescence staining of testicular samples is reported in our recent publications (12, 13, 31). The information for primary antibodies is listed in Table S1.

### Statistical analysis.

Data were analyzed using SPSS statistical software (IBM Co., NY) with one-way analysis of variance (ANOVA) followed by LSD multiple-comparison tests. All groups were compared with each other for every parameter. The data were shown as the mean ± SEM. Statistical significance was based on *P* < 0.05.

### Data availability.

Liver sample RNA-seq raw data are deposited in NCBI’s Gene Expression Omnibus under accession number GSE175881. The microbiota raw sequencing data generated in this study has been uploaded to the NCBI SRA database with the accession numbers PRJNA734312 (small intestine), PRJNA734318 (cecum), and PRJNA734334 (colon).
