# Supplementary material for: Gut Microbiota-Testis Axis: FMT Mitigates High-Fat Diet-Diminished Male Fertility via Improving Systemic and Testicular Metabolome
Source: Microbiol Spectr. 2022 Apr 21;10(3):e00028-22. doi: 10.1128/spectrum.00028-22 (PMC9241630; doi:10.1128/spectrum.00028-22)
Supplement: SUPPLEMENTAL FILE 1 — Supplemental material. Download spectrum.00028-22-s001.pdf, PDF file, 1.1 MB [file spectrum.00028-22-s001.pdf]

Supplementary Materials

Detailed Methods

Evaluation of spermatozoa motility using a computer-assisted sperm analysis system

Spermatozoa motility was assessed using a computer-assisted sperm assay (CASA) method according to World Health Organization guidelines [1]. After euthanasia, spermatozoa were collected from the cauda epididymis of mice and suspended in DMEM/F12 medium with 10% FBS and incubated at 37.5 °C for 30 min; samples were then placed in a pre-warmed counting chamber. The microscopic sperm class analyzer (CASA system) was used in this investigation. It was equipped with a 20-fold objective, a camera adaptor (Eclipse E200, Nikon, Japan), and a camera (acA780-75gc, Basler, Germany), and it was operated by an SCA sperm class analyzer (MICROPTIC S.L.). The classification of sperm motility was as follows: grade A linear velocity >22 μm s^{-1}; grade B <22 μm s^{-1} and curvilinear velocity >5 μm s^{-1}; grade C curvilinear velocity <5 μm s^{-1}; and grade D = immotile spermatozoa. The spermatozoa motility data represented only grade A + grade B since only these two grades are considered to be functional.

Morphological observations of spermatozoa

*T*he extracted murine caudal epididymides were placed in RPMI medium, finely chopped, and then Eosin Y (1%) was added for staining as described previously [1]. Spermatozoon abnormalities were then viewed using an optical microscope and were classified into head or tail morphological abnormalities: two heads, two tails, blunt hooks, and short tails. The examinations were repeated three times, and 500 spermatozoa per animal were scored.

Assessment of acrosome integrity

After harvest, mouse spermatozoa were incubated at 37.5 °C for 30 min, after which a

drop of sperm suspension was uniformly smeared on a clean glass slide. Smeared slides were air dried and incubated in methanol for 2 min for fixation. After fixation, the slides were washed with PBS three times. Assessment of an intact acrosome was accomplished by staining the sperm with 0.025% Coomassie brilliant blue G-250 in 40% methanol for 20 min at room temperature (RT). The slides were then washed three times with PBS and mounted with 50% glycerol in PBS. Acrosomal integrity was determined by an intense staining on the anterior region of the sperm head under bright-field microscopy (AH3-RFCA, Olympus, Tokyo, Japan) and scored accordingly [1].

RNA Isolation and RNA-seq analyses [1]

Briefly, total RNA was isolated using TRIzol Reagent (Invitrogen) and purified using a Pure-Link1 RNA Mini Kit (Cat: 12183018A; Life Technologies) following the manufacturers' protocol. Total RNA samples were first treated with DNase I to degrade any possible DNA contamination. Then the mRNA was enriched using oligo(dT) magnetic beads. Mixed with the fragmentation buffer, the mRNA was broken into short fragments (about 200 bp), after which, the first strand of cDNA was synthesized using a random hexamer-primer. Buffer, dNTPs, RNase H, and DNA polymerase I were added to synthesize the second strand. The double strand cDNA was purified with magnetic beads. Subsequently, 3'-end single nucleotide A (adenine) addition was performed. Finally, sequencing adaptors were ligated to the fragments. The fragments were enriched by PCR amplification. During the QC step, an Agilent 2100 Bioanaylzer and ABI StepOnePlus Real-Time PCR System were used to qualify and quantify the sample library. The library products were prepared for sequencing in an Illumina HiSeqTM 2500. The reads were mapped to reference genes using SOAPaligner (v. 2.20) with a maximum of two nucleotide mismatches allowed at the

parameters of “-m 0 -x 1000 -s 40 -l 35 -v 3 -r 2”. The read number of each gene was transformed into RPKM (reads per kilo bases per million reads), and then differentially expressed genes were identified using the DEGseq package and the MARS (MA-plot-based method with random sampling model) method. The threshold was set as FDR ≤0.001 and an absolute value of $\log_2$ ratio ≥1 to judge the significance of the difference in gene expression. Then on the data were analyzed by GO enrichment, KEGG enrichment.

Sequencing of microbiota from intestine digesta samples and data analysis [1]

DNA Extraction. Total genomic DNA of small intestine, cecum and colon digesta was isolated using an E.Z.N.A.R Stool DNA Kit (Omega Bio-tek Inc., USA) following the manufacturer’s instructions. DNA quantity and quality were analyzed using NanoDrop 2000 (Thermo Scientific, USA) and 1% agarose gel. Ten samples/groups were determined. ***Library preparation and sequencing.*** The V3-V4 region of the 16S rRNA gene was amplified using the primers MPRK341F (50-ACTCCTACGGGAGGCAGCAG -30) and MPRK806R: (50-GGACTACHVGGGTWTCTAAT -30) with Barcode. The PCR reactions (total 30 μL) included 15 μL PhusionR High-Fidelity PCR Master Mix (New England Biolabs), 0.2 mM primers, and 10 ng DNA. The thermal cycle was carried out with an initial denaturation at 98 °C, followed by 30 cycles of 98 °C for 10 s, 50 °C for 30 s, 72 °C for 30 s, and a final extension at 72 °C for 5 min. PCR products were purified using a GeneJET Gel Extraction Kit (Thermo Scientific, USA). The sequencing libraries were constructed with NEB NextR UltraTM DNA Library Prep Kit for Illumina (NEB, United States) following the manufacturer’s instructions and index codes were added. Then, the library was sequenced on the Illumina HiSeq 2500 platform and 300 bp paired-end reads were generated at the Novo gene. The paired-end reads were merged

using FLASH (V1.2.71). The quality of the tags was controlled in QIIME (V1.7.02), meanwhile all chimeras were removed. The "Core Set" of the Greengenes database3 was used for classification, and sequences with >97% similarity were assigned to the same operational taxonomic units (OTUs). ***Analysis of sequencing data*** Operational taxonomic unit abundance information was normalized using a standard of sequence number corresponding to the sample with the least sequences. The alpha diversity index was calculated with QIIME (Version 1.7.0). The Unifrac distance was obtained using QIIME (Version 1.7.0), and PCoA (principal coordinate analysis) was performed using R software (Version 2.15.3). The linear discriminate analysis effect size (LEfSe) was performed to determine differences in abundance; the threshold LDA score was 4.0. GraphPad Prism7 software was used to produce the graphs.

Plasma and testis metabolite measurements by LC-MS/MS

Plasma samples were collected and immediately stored at -80 °C. Before LC-MS/MS analysis, the samples were thawed on ice and processed to remove proteins. Testis samples were collected and the same amount of tissue from each mouse testis was used to isolate the metabolites using CH_3OH: H_2O (V: V) = 4:1. Then samples were detected by ACQUITY UPLC and AB Sciex Triple TOF 5600 (LC/MS) as reported previously [1,2]. Fifteen samples/groups were analyzed for plasma or testis samples. The HPLC conditions employed an ACQUITY UPLC BEH C18 column (100 mm × 2.1 mm, 1.7 μm), solvent A [aqueous solution with 0.1% (v/v) formic acid], and solvent B [acetonitrile with 0.1% (v/v) formic acid] with a gradient program. The flow rate was 0.4 mL/min and the injection volume was 5 μL. Progenesis QI v2.3 (Nonlinear Dynamics, Newcastle, UK) was implemented to normalize the peaks. Then the Human Metabolome Database (HMDB), Lipidmaps (v2.3), and METLIN software were used to qualify the data. Moreover, the data were processed with

SIMCA software (version 14.0, Umetrics, Umeå, Sweden) following pathway enrichment analysis using the KEGG database (http://www.genome.jp/KEGG/pathway.html).

Liver oil red staining

The fresh liver tissue was washed twice with PBS. Then the tissue was incubated in 15% sucrose (in PBS) for 15 min and transferred to 30% sucrose (in PBS) for 15 min. Subsequently, the tissue was embedded in an optimal cutting temperature compound for frozen cell specimens. Further, the sections were cut into 5 µm- using a freezing microtome and sections were washed in PBS three times. The sections were subsequently stained using an Oil Red O staining kit according to the manufacturer's instructions (Cat No.: D027; Nanjing Jiancheng Bioengineering Institute, Nanjing, P.R. China) [3, 4].

Determination of blood TG, TC and T-AOC

Blood TG, TC and T-AOC were determined by the kits from Nanjing Jiancheng Bioengineering Institute [Nanjing, P.R. China; TG (Cat. #: A110-1-1); TC (Cat. #: A111-1-1); T-AOC (Cat. #: A015-2-1)] [4]. All the procedures were followed from the manufacturer's instructions.

Qualification of liver retinol and retinol acids by HPLC

The liver content of retinol and retinol acid were determined followed the reported methods by HPLC [5].

Histopathological analysis

Testicular tissues were fixed in 10% neutral buffered formalin, paraffin embedded, cut into 5 µm sections and subsequently stained with hematoxylin and eosin (H&E) for histopathological analysis.

Western blotting

Western blotting analysis of proteins was carried out as previously reported [1,2,6]. Briefly, testicular tissue samples were lysed in RIPA buffer containing the protease inhibitor cocktail from Sangong Biotech, Ltd. (Shanghai, China). Protein concentration was determined using a BCA kit (Beyotime Institute of Biotechnology, Shanghai, China). Goat anti-actin was used as a loading control. The information for primary antibodies (Abs) were listed in online Table S1. Secondary donkey anti-goat Ab (Cat no.: A0181) was purchased from Beyotime Institute of Biotechnology, and goat anti-rabbit (Cat no.: A24531) Abs were bought from Novex® by Life Technologies (USA). Fifty micrograms of total protein per sample were loaded onto 10% SDS polyacrylamide electrophoresis gels. The gels were transferred to a polyvinylidene fluoride (PVDF) membrane at 300 mA for 2.5 h at 4 °C. The membranes were then blocked with 5% bovine serum albumin (BSA) for 1 h at RT, followed by three washes with 0.1% Tween-20 in TBS (TBST). The membranes were incubated with primary Abs diluted with 1:500 in TBST with 1% BSA overnight at 4 °C. After three washes with TBST, the blots were incubated with the HRP-labelled secondary goat anti-rabbit or donkey anti-goat Ab respectively for 1 h at RT. After three washes, the blots were imaged. The bands were quantified using Image-J software. The intensity of the specific protein band was normalized to actin first, then the data were normalized to the control. The experiment was repeated >6 times.

Detection of protein levels and location in testis using immunofluorescence staining

The methodology for immunofluorescence staining of testicular samples is reported in our recent publications [1,2,6]. Sections of testicular tissue (5 μm) were prepared and subjected to antigen retrieval and immunostaining as previously described. Briefly, sections were first blocked with normal goat serum in PBS, followed by incubation with primary Abs (Table S1; 1:100 in PBS-0.5% Triton X-100; Bioss Co. Ltd. Beijing,

PR China) at 4 °C overnight. After a brief wash, sections were incubated with an Alexa 546-labeled goat anti-rabbit secondary Ab (1:100 in PBS; Molecular Probes, Eugene, OR, USA) at RT for 30 min and then counterstained with 4',6-diamidino-2-phenylindole (DAPI). The stained sections were examined using a Leica Laser Scanning Confocal Microscope (LEICA TCS SP5 II, Germany). Ten animal samples from each treatment group were analysed. Positively stained cells were counted. A minimum of 1000 cells were counted for each sample of each experiment. The data were then normalized to the control.

References:

1. Zhao, Y., Zhang, P., Ge, W., Feng, Y., Li, L., Sun, Z., Zhang, H., Shen, W. Alginate oligosaccharides improve germ cell development and testicular microenvironment to rescue busulfan disrupted spermatogenesis. *Theranostics*. 2020; 10: 3308-3324.
2. Zhang, P., Feng, Y., Li, L., Ge, W., Yu, S., Hao, Y., Shen, W., Han, X., Ma, D., Yin, S., et al. Improvement in sperm quality and spermatogenesis following fecal microbiota transplantation from alginate oligosaccharide dosed mice. *Gut* 2021; 70: 222-225.
3. Xiong, B., Liu, M., Zhang, C., Hao, Y., Zhang, P., Chen, L., Tang, X., Zhang, H., Zhao, Y. Alginate oligosaccharides enhance the small intestine cell integrity and migration ability. *Life Sci.* 2020; 258: 118085.
4. Chu, M., Zhao, Y., Yu, S., Hao, Y., Zhang, P., Feng, Y., Zhang, H., Ma, D., Liu, J., Cheng, M., et al. MicroRNA-221 may be involved in lipid metabolism in mammary epithelial cells. *Int. J. Biochem. Cell Biol.* 2018; 97: 118-127.
5. Elias-Miró, M., Massip-Salcedo, M., Raila, J., Schweigert, F., Mendes-Braz, M., Ramalho, F., Jiménez-Castro, M.B., Casillas-Ramírez, A., Bermudo, R., Rimola, A., Rodes, J., et al. Retinol binding protein 4 and retinol in steatotic and nonsteatotic rat livers in the setting of partial hepatectomy under ischemia/reperfusion. *Liver Transpl.* 2012; 18: 1198-208.
6. Zhang, C., Xiong, B., Chen, L., Ge, W., Yin, S., Feng, Y., Sun, Z., Sun, Q., Zhao, Y., Shen, W., Zhang, H. Rescue of male fertility following fecal microbiota transplantation from alginate oligosaccharide dosed mice. *Gut* 2020; doi: 10.1136/gutjnl-2020-323593.

Supplementary Information

Supplemental Figures

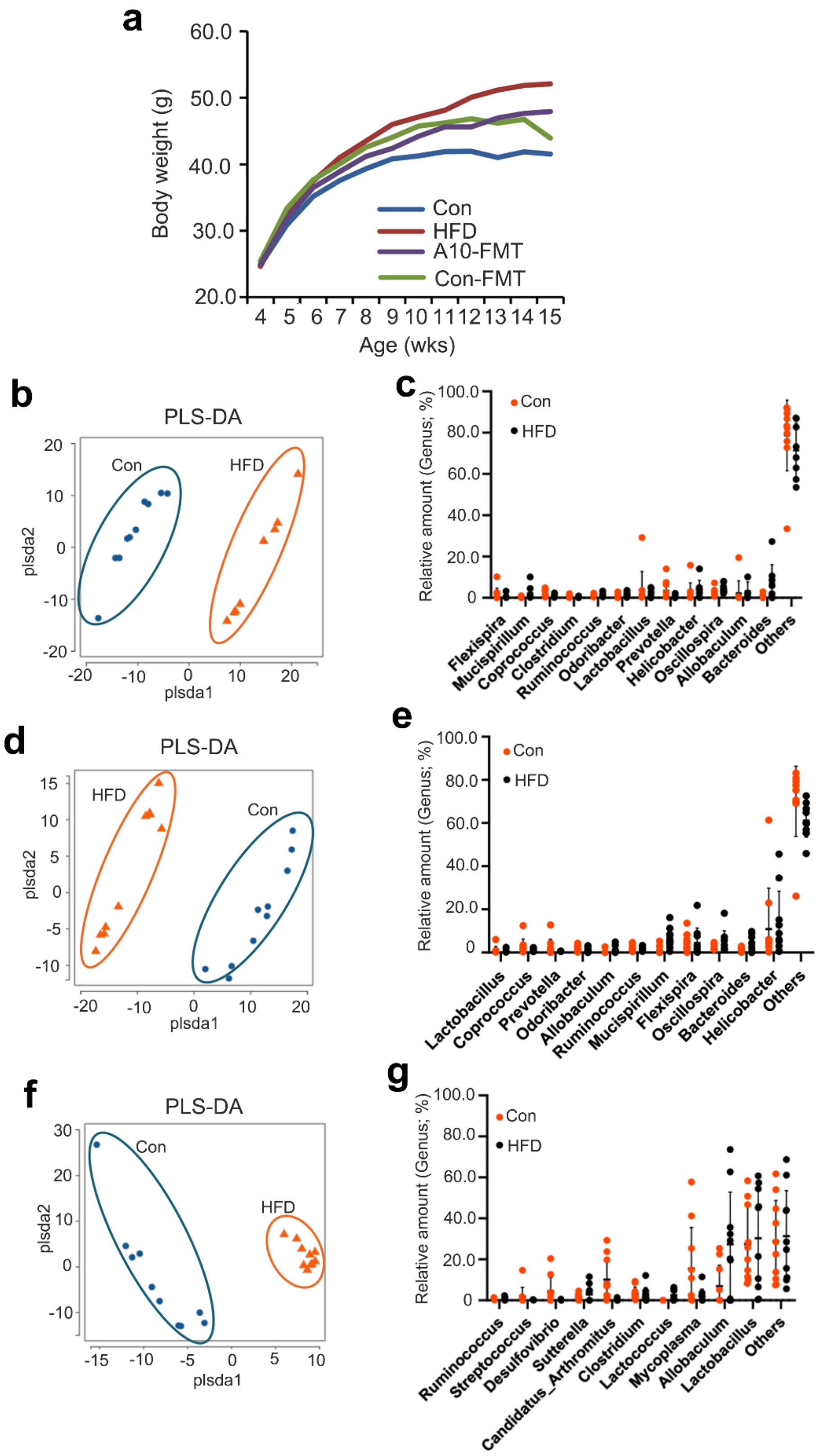

Figure S1. Body weight and gut microbiota changes (HFD vs. Con). **(a)** Animal

bodyweight in the long-term experiment in different treatment. The y-axis represents the body weight (g). The x-axis represents the age (weeks). **(b)** PLS-DA (OTU) of colon microbiota in HFD and Con groups. **(c)** Colon microbiota levels at genus level in HFD and Con groups. The y-axis represents the relative amount (%). The x-axis represents the individual microbiota. **(d)** PLS-DA (OTU) of cecum microbiota in HFD and Con groups. **(e)** Cecum microbiota levels at genus level in HFD and Con groups. The y-axis represents the relative amount (%). The x-axis represents the individual microbiota. **(f)** PLS-DA (OTU) of small intestine microbiota in HFD and Con groups. **(g)** Small intestine microbiota levels at genus level in HFD and Con groups. The y-axis represents the relative amount (%). The x-axis represents the individual microbiota.

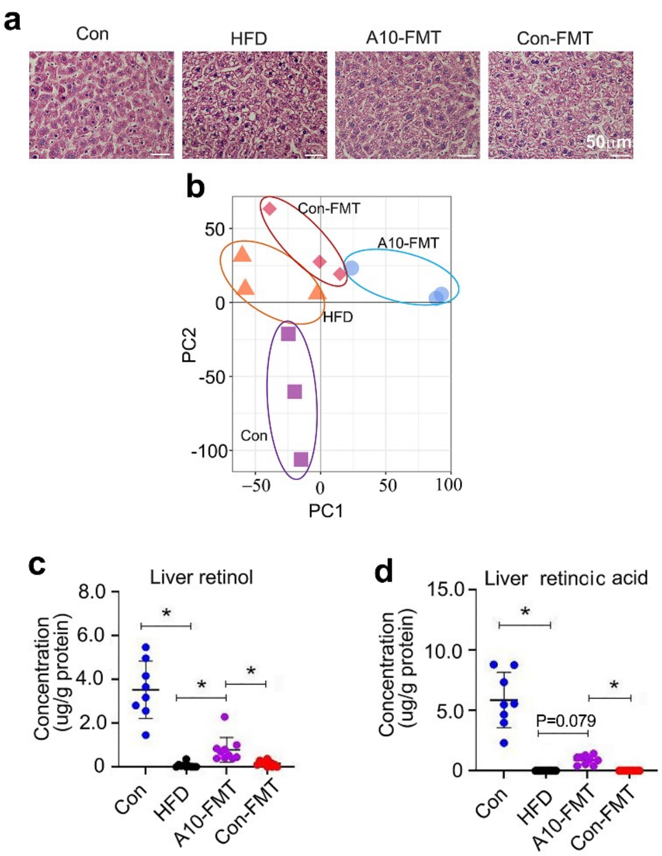

Figure S2. Liver lipid metabolism changes, and cholic acids and retinol signaling involvement. **(a)** Histopathology staining (HE) of liver samples in each treatment. Scale bar: 50μm. **(b)** Summary of RNA-seq analysis of liver samples for each treatment (PCA). **(c)** Liver retinol levels (by HPLC quantification) in each treatment. The y-axis represents the concentration. The x-axis represents the treatment. $^{*}p < 0.05$. **(d)** Liver retinoic acid levels (by HPLC quantification) in each treatment. The y-axis represents the concentration. The x-axis represents the treatment. $^{*}p < 0.05$.

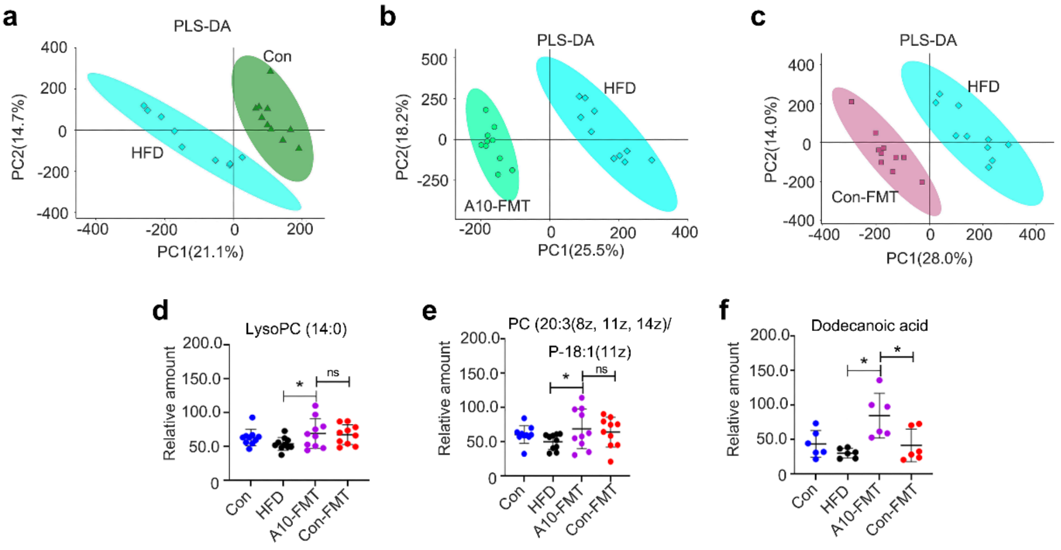

Figure S3. Blood metabolite changes among Con, HFD, A10-FMT and Con-FMT. **(a)** PLS-DA of blood metabolites in Con and HFD groups. **(b)** PLS-DA of blood metabolites in HFD and A10-FMT groups. **(c)** PLS-DA of blood metabolites in HFD and Con-FMT groups. **(d)** Blood LysoPC (14:0) levels (by HPLC quantification) in each treatment. The y-axis represents the concentration. The x-axis represents the treatment. $^{*}p < 0.05$. **(e)** Blood PC (20:3(8z, 11z, 14z)/P-18:1(11z) levels (by HPLC quantification) in each treatment. The y-axis represents the concentration. The x-axis represents the treatment. $^{*}p < 0.05$. **(f)** Blood Dodecanoic acid levels (by HPLC quantification) in each treatment. The y-axis represents the concentration. The x-axis represents the treatment. $^{*}p < 0.05$.

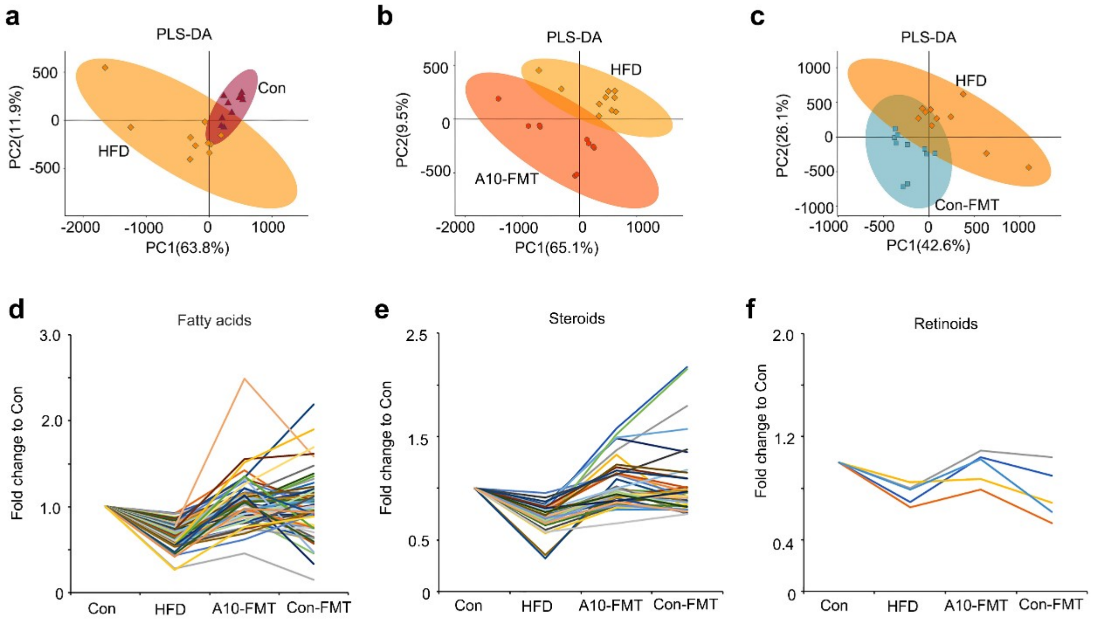

Figure S4. Testicular metabolite changes among Con, HFD, A10-FMT and Con-FMT. **(a)** PLS-DA of testicular metabolites in Con and HFD groups. **(b)** PLS-DA of testicular metabolites in HFD and A10-FMT groups. **(c)** PLS-DA of testicular metabolites in HFD and Con-FMT groups. **(d)** Summary of the testicular changed fatty acids (by HPLC quantification) in each treatment. **(e)** Summary of the testicular changed steroids (by HPLC quantification) in each treatment. **(f)** Summary of the testicular changed retinoids (by HPLC quantification) in each treatment.

Supplementary Tables

Table S1. Primary antibody information.

Supplementary Data Sets

Data Set 1. Blood metabolites raw data.

Data Set 2. Testicular metabolites raw data.

Table S1. Primary antibody information

Gene symbol	Name	Cat. #	Predicted size	Source (Animal)	Company
DDX4 (VASA)	DEAD (Asp Glu Ala Asp) box polypeptide	ab13840	76kDa	Rabbit (polyclonal)	Abcam
DAZL	DAZ like autosomal	ab34139	33kDa	Rabbit (polyclonal)	Abcam
SCP3/SYCP3	Synaptonemal complex protein 3	NB300-232	28kDa	Rabbit (polyclonal)	Novus Biologicals
SOX9	SRY (sex-determining region Y)-box 9 protein	AB5535	65kDa	Rabbit (polyclonal)	Merck Millipore
TNP1(TP1)	Transition protein-1	ab73135		Rabbit (polyclonal)	Abcam
StAR	Steroidogenic acute regulatory protein	bs-3570R	32kDa	Rabbit (polyclonal)	Beijing Biosynthesis Biotechnology CO.
CYP11A1	Cholesterol side chain cleavage enzyme	bs-10099R	53/57kDa	Rabbit (polyclonal)	Beijing Biosynthesis Biotechnology CO.
PIWIL1	Piwi like protein 1	ab94917	99kDa	Rabbit (polyclonal)	Abcam
Prm2	Protamine 2	bs-6371R	7kDa	Rabbit	Beijing Biosynthesis Biotechnology CO.
PGK2	Phosphoglycerate kinase 2	D121803	45kDa	Rabbit (polyclonal)	Sangon Biotech (Shanghai) Co., Ltd.
Acrosin	Anti-Acrosin antibody	Ab-203289	46kDa	Rabbit	Abcam
p-GSK3a	Glycogen synthase kinase-3 alpha (Ser21)	bs-4692R	54kDa	Rabbit (polyclonal)	Beijing Biosynthesis Biotechnology CO.
FXR	Bile Acid Receptor (NNR1H4)	Bs-12867R	56kDa	Rabbit (polyclonal)	Beijing Biosynthesis Biotechnology CO.
DHRS9	Dehydrogenase/reductase SDR family member 9	Bs-7859R	35kDa	Rabbit (polyclonal)	Beijing Biosynthesis Biotechnology CO.
RBP4	Retinol binding protein 4	D122994-0025	23kDa	Rabbit (polyclonal)	Sangon Biotech (Shanghai) Co., Ltd.
HSP70	Heat shock 70 kDa protein	bs-0126R	70kDa	Rabbit (polyclonal)	Beijing Biosynthesis Biotechnology CO.
SOD1	Super oxide dismutase	bs-1080R	22kDa	Rabbit (polyclonal)	Beijing Biosynthesis Biotechnology CO.
GPX1	Glutathione peroxidase 1	bs-3882R	22kDa	Rabbit (polyclonal)	Beijing Biosynthesis Biotechnology CO.
actin	actin	Ab3280	42kDa	Rabbit (polyclonal)	Abcam
